# Evidence for FOXL2 Association with the Tsc1 Regulatory Region in Mice

**DOI:** 10.3390/biom16040510

**Published:** 2026-03-29

**Authors:** Mara Marongiu, Loredana Marcia, Andrea Sbardellati, Manila Deiana, Isadora Asunis, Emanuele Pelosi, Andrea Coschiera, Francesca Crobu, Angela Loi, Emilio Melis, Maria Cristina Mostallino, Alessandra Meloni, Roberto Cusano, Francesco Cucca, Manuela Uda, Laura Crisponi

**Affiliations:** 1 Institute for Genetic and Biomedical Research, National Research Council, 09042 Monserrato, Italy; 2 Centre for Advanced Studies, Research and Development in Sardinia (CRS4), Science and Technology Park Polaris, 09050 Pula, Italy; 3 National Institute on Aging, National Institute of Health, DHHS, Baltimore, MD 21224, USA; 4 Institute of Neuroscience, National Research Council, 09042 Monserrato, Italy; 5 Department of Biomedical Science, University of Sassari, 07100 Sassari, Italy

**Keywords:** FOXL2, *Tsc1*, mTORC pathway, ovarian reserve, menopause, fertility

## Abstract

Ovarian reserve and reproductive life are closely linked concepts in female reproductive biology. The ovarian reserve consists of primordial follicles and refers to the number and quality of oocytes (eggs) remaining in the ovaries at any given time. Follicular dynamics shape a woman’s reproductive lifespan, ultimately leading to menopause. Elucidating the underlying genetic and molecular pathways of follicle maturation and depletion is thus crucial for understanding menopausal onset and progression, both in normal and pathophysiological contexts, such as primary ovarian insufficiency, defined as menopause before the age of 40. A key factor in ovarian differentiation and fertility maintenance is FOXL2, a forkhead family transcription factor that plays a crucial role in follicle formation and development, ovarian maintenance, and sex determination. By employing a ChIP-Seq approach in mice, we identified a previously unreported binding of FOXL2 to a *Tsc1* regulatory region. Our data, along with a thorough literature review, support the hypothesis that FOXL2-mediated activation of *Tsc1* in granulosa cells can help maintain primordial follicles in a dormant state by suppressing mTORC1 signalling. Understanding the mechanisms behind ovarian reserve may lay the foundation for developing novel fertility preservation strategies, improving fertility treatment protocols and promoting in vitro activation of cryopreserved ovarian tissue to support folliculogenesis.

## 1. Introduction

The Forkhead Box L2 (FOXL2) transcription factor is a critical regulator of female reproductive longevity, governing the transition of follicles from dormancy to maturation. In modern clinical contexts, understanding these molecular mechanisms is increasingly vital, as delayed childbearing beyond the age of 30 has made ovarian ageing and the depletion of the ovarian reserve a significant public health concern.

In mice, FOXL2 expression is activated in the female gonads as early as 12.5 dpc and persists from the primordial stage through the maturing follicle stages. Primordial follicles are the basic reproductive units in female mammals, consisting of a germ cell (immature oocyte) surrounded by a single layer of somatic primordial follicle granulosa cells (pfGCs). The pool of dormant primordial follicles represents the ovarian reserve, remaining quiescent until activated by a tightly regulated cascade of molecular and cellular events. The activation triggers follicular growth and the awakening of oocytes, ultimately leading to ovulation. The process is characterised by an increase in oocyte size and the transition of the surrounding pre-granulosa cells from a flattened/squamous to cuboidal form [1,2]. The length of a female’s reproductive lifespan thus depends on the size of this reserve and the rate at which follicles are activated. Once the reserve is exhausted through ovulation and cell loss, menopause occurs. FOXL2 prevents premature activation and supports the survival and identity of follicles once they begin to grow. When the FOXL2 function is compromised, the narrative of follicular development is disrupted, leading to clinical pathologies. In humans, mutations in the *FOXL2* gene can lead to primary ovarian insufficiency (POI) in women and eyelid abnormalities in both genders [blepharophimosis-ptosis-epicanthus inversus syndrome (BPES, MIM#110100) [3,4]. *Foxl2* homozygous knock-out (KO; *Foxl2^−/−^*) mice recapitulate features of human BPES, displaying eyelid hypoplasia and female infertility [5,6]. The experimental data from these mice provide insight into the cellular failures caused by FOXL2 deficiency. From P0 to adulthood, pfGCs remain in a flat, squamous state and fail to transition to the cuboidal form, leading to differentiation arrest. Oocytes remain trapped in nests because the necessary breakdown of cysts is inhibited [5,6,7]. Two weeks after birth, nearly all oocytes in *Foxl2^−/−^* ovaries express GDF9 and KITL, indicating their activation and the beginning of folliculogenesis [5,6,8]. Despite this, the GDF9 signal, which promotes the progression of early primary follicles by also promoting FOXL2 expression [9], is impaired because granulosa cells lacking FOXL2 remain flat and lose identity [10]. Mutants, in the absence of functional granulosa cells, exhibit uncontrolled oocyte growth, leading to atresia, follicular depletion, and infertility [5,6,8]).

The mTORC1 and PI3K/Akt signalling pathways also play key roles in follicle maturation. In the pfGCs, the mTORC1 pathway operates upstream of PI3K/Akt, and it is negatively regulated by a heterodimeric complex composed of TSC1 and TSC2 [11]. mTORC1 signalling triggers primordial follicle activation by increasing KITL expression in pfGCs. KITL then binds to cKIT on oocyte surfaces, activating downstream PI3K/Akt signalling [12]. This activation leads to Akt phosphorylation, which in turn phosphorylates FOXO3 within the oocyte, causing its relocation from the nucleus to the cytoplasm and thereby promoting follicle activation [12,13,14].

In parallel, the activated PI3K/Akt signalling pathway leads to phosphorylation and inactivation of the TSC1/2 complex in the oocyte, thereby releasing its inhibitory control over mTORC1 and promoting activation pathway [15,16].

In the context of folliculogenesis, mTORC1 activity, mediated by TSC1, is essential for early follicular growth and progression beyond the primordial stage [12,17,18,19]. In concert, these pathways form a finely tuned network that determines the fate choice of a follicle whether it remains dormant or begins to grow. Alterations in these pathways may thus contribute to cases of female infertility due to primordial follicle depletion, including POI and primary amenorrhea [17,20]. The mTORC1 and PI3K/Akt pathways are primarily important during early folliculogenesis, as they are largely independent of gonadotropins. However, as they mature into primary and secondary follicles, the follicle-stimulating hormone (FSH), produced by gonadotropes in the anterior pituitary in response to activins and FOXL2 [21,22], becomes critically involved in granulosa cell development and estradiol synthesis, with its levels indicative of ovarian reserve; elevated FSH levels may signal decreased reserve, whereas lower levels reflect better follicular levels [23,24].

Thus, to further explore FOXL2’s role in ovarian function and fertility maintenance, and in light of the Hypothalamic–Pituitary–Gonadal (HPG) axis, we used a ChIP-Seq approach to identify FOXL2 target genes in P7 mouse ovaries and in the Alpha T3-1 cell line, a well-established model derived from pituitary gonadotrope cells that express FOXL2 [25,26]. In both experiments, we identified a shared binding site at the *Tsc1* locus and confirmed the motif’s specificity. This evidence supports the hypothesis that FOXL2, potentially regulating *Tsc1* transcription in granulosa cells, may contribute to maintaining primordial follicles dormant by limiting mTORC1 signalling.

## 2. Materials and Methods

### 2.1. Animal Care

All animal experiments adhered to the ARRIVE guidelines (https://www.nc3rs.org.uk/arrive-guidelines, last access in 13 February 2026) and were conducted in accordance with the EU Directive 2010/63/EU for the protection of animals used in scientific research (http://ec.europa.eu/environment/chemicals/lab_animals/legislation_en.htm, last access in 13 February 2026). *Foxl2^−/−^* mice were generated by deleting the entire *Foxl2* coding region, as previously described [5]. C57BL/6 mice were used for this study. The mice were housed conventionally at a constant temperature (20–24 °C) and humidity (50–60%) in an animal facility with a 12 h light–dark cycle, with free access to food and water. The mice were sacrificed through decapitation. PCR genotyping reactions with specific primers [5] were used to detect wild-type (WT) and mutant alleles.

### 2.2. Chromatin Immunoprecipitation Sequencing (ChIP-Seq)

The ChIP technique followed the protocol outlined by Dahl et Collas, 2008 [27]. ChIP-Seq was performed on ovaries from *Foxl2* WT P7 mice (*n* = 3) and the mouse pituitary Alpha T3-1 cell line (10^9^ cells for each experiment, *n* = 3), as previously reported [28]. Both ChIP-Seq and qPCR, conducted on mouse ovaries and Alpha T3-1 cells, included three biological and three technical replicates. For immunoprecipitation, we used a custom-made anti-FOXL2 rabbit polyclonal antibody raised against the C-terminal residues, DHDSKTGALHSRLDL (Kaneka Eurogentec S.A., Seraing, Belgium). Antibody specificity was previously confirmed through Western blotting and was used in several studies from our group [5,28,29]. The negative control (mock IP) was prepared by performing the entire immunoprecipitation protocol using a non-specific IgG antibody instead of the anti-FOXL2-specific antibody.

To validate the ChIP experiments prior to sequencing, we used ChIP-qPCR to assess the enrichment of the known FOXL2 target gene, *StAR*, in the immunoprecipitated sample [30] in both experiments. The primers used were sense 5′ CCCCTGCTTTCCCCTACCT 3′, and anti-sense 5′ TGGGAGGGAGCAGACTGTGT 3′ (Supplementary Figure S1).

For ChIP-Seq, paired-end libraries were prepared following standard Illumina protocols, with size selection for 200-nucleotide inserts. The sequencing was performed on an Illumina Genome Analyzer II (Illumina, Inc., San Diego, CA, USA) to generate 21 nt paired-end reads. The reads were mapped to the mm9 reference genome using Bowtie [31] in paired-end mode with zero mismatches. MACS [32] identified potential FOXL2-binding regions, analysing samples and controls separately. ChIP regions were annotated with CEAS [33] based on RefSeq. The regions within 0–1000 nt upstream of the transcription start site (TSS) were used for motif analysis via MEME-ChIP [34]. MEME-ChIP (http://meme.sdsc.edu/meme/ accessed in 1 March 2014) and CisFinder (http://lgsun.grc.nia.nih.gov/CisFinder/, accessed in 1 March 2014) were used to find over-represented short DNA motifs in the ChIP-Seq data. We also conducted a second independent analysis in 2022 with an overlapping updated pipeline, through the commercial service, GalSeq srl (Milan, Italy). We chose to retain the same mm9 reference genome to ensure data comparability with our previous analysis [28].

The peak-calling lower threshold was set at a q-value (−log10) of 1.3, while a very highly significant level was considered at a q-value (−log10) of 6, which corresponds to a *p*-value (−log10) of 10. To remove the background noise, the input served as a control. It consists of cross-linked DNA processed as a ChIP sample for DNA extraction, but it omits the immunoprecipitation step.

### 2.3. ChIP–Quantitative Polymerase Chain Reaction (ChIP-qPCR)

To confirm Tsc1 binding, we repeated the ChIP experiments in the *Foxl2* WT P7 mice (*n* = 3) and in the mouse pituitary Alpha T3-1 cell line (10^9^ cells for each experiment, *n* = 3), followed by qPCR. ChIP-qPCR was performed to confirm FOXL2 binding to *Tsc1* using specific primer pairs: *Tsc1* sense 5′ GGAGGAGACGGTGGGTGAGT 3′ and anti-sense 5′ CCCCGAAAAGCCCACAA 3′, along with a negative control sense 5′ GGGCTTGGCTCTGGTAGGA 3′ and anti-sense 5′ CTAGCTTCAAAGCCCTGATGGT 3′. The qPCR mix contained 1 μL of a previously five-fold-diluted ChIP preparation, 2.5 μM of each primer, and 2× Universal TaqMan master mix (Life Technologies Applied Biosystems, Carlsbad, CA, USA) to a total volume of 10 μL. The dissociation curves confirmed the specificity of the amplicons and the absence of primer dimer contamination. RT-qPCR was conducted using the ABI PRISM^®^ 7700 Sequence Detection System (Applied Biosystems, Foster City, CA, USA). Quantitative PCR was performed on three biological replicates of mouse ovaries and Alpha T3-1 cells, with each sample analysed in three technical replicates.

Fold enrichment relative to the mock control was calculated based on threshold cycle (CT) measurements and the delta–delta Ct method (2−ΔΔCt), where ΔΔCt equals (Ct IP) − (Ct mock).

### 2.4. Pathway and Process Enrichment Analysis

To perform gene ontology and pathway analysis, we used gene lists from significant GalSeq peaks. Each list was analysed for enrichment using Metascape (https://metascape.org/, accessed in 1 July 2025). For each gene list, pathway, and process, an enrichment analysis was conducted with the following ontology sources: GO Biological Processes, KEGG Pathway, Reactome Gene Sets, CORUM, WikiPathways, and PANTHER Pathway. All genes in the genome served as the background for enrichment analysis. In Metascape, ‘enrichment’ shows how many-fold more pathway members are present in our gene list than would be expected by chance. The *p*-value (log10P) is the most commonly used metric [35].

An automatic/express analysis was performed in July 2025, with Metascape 3.5, Analysis Database, version v3.5.20260201 (see https://metascape.org/gp/index.html#/menu/release_history, last accessed in 1 March 2026, for detailed information).

### 2.5. Electrophoretic Mobility Shift and Supershift Analysis (EMSA)

Small-scale nuclear extracts were prepared from Alpha T3-1 cells, transfected with either 2.5 μg of the empty vector or pCRUZ-Myc-*Foxl2*:WT. EMSAs followed the original protocol [36], with minor modifications: 10 μg of protein lysate were incubated for 20 min at 25 °C with 10 fmoles/30,000 cpm of a T4 kinase/32P-γ ATP labelled double-stranded probe. These reactions were performed in 5% acrylamide gels (50/1 cross-linking) using 50 mmol/L Tris borate buffer at 10 V/cm, then dried and autoradiographed overnight at −80 °C. The sense strand of the 30 bp oligonucleotide probe containing the WT consensus motif was 5′ GGGACTGTGAGGTAAACAGCTGAGGGGGAG 3′, whereas the mutant (MUT) probe was 5′ GGGACTGTGAGGTCCAGAGCTGAGGGGGAG 3′. For the supershift assay, the EMSA reactions were pre-incubated for 20 min at room temperature with rabbit polyclonal anti-FOXL2, raised against residues FRPPPAHFQPGKGLF (Eurogentec s.a., Belgium), within the forkhead DNA-binding domain, and affinity-purified. Specificity was confirmed through Western blotting. This antibody has been extensively validated in previous studies from our group [5,28,29]. The experiments were conducted in triplicate to ensure reliability. The mutated probe served as a negative control.

### 2.6. Transactivation Assays

We used constructs containing 755 bases of the *Tsc1* locus, which includes two FoxO-response elements (FREs) cloned upstream of the firefly cDNA (*Tsc1*:FRE^WT^ and *Tsc1*:FRE^MUT^) in pGL2 Luciferase Reporter Vector (Promega, Madison, WI, USA). These constructs are the same as those reported in the paper by Khatri et al., 2010 [37]. In the *Tsc1*:FRE^MUT^ construct, three nucleotides from the core of the FoxO consensus binding site of each FRE were mutated: FRE1, TTGTTT to TTCTGG, and FRE2, AAACAG to CCAGAG. The FRE2 -AAACAG- corresponds to the FOXL2-bound region -GTAAACA- identified by ChIP-Seq and matches our MUT region used in the EMSA experiment. Transfection was performed in HEK293 cells using Lipofectamine LTX (Invitrogen, Thermo Fisher Scientific Inc., Waltham, MA, USA), with 1 µg of either *Tsc1*:FRE^WT^ or *Tsc1*:FRE^MUT^ luciferase reporter construct, along with 2 µg of mouse pCRUZ-Myc-*Foxl2*:WT.

A Renilla Luciferase control vector (Promega, Madison, WI, USA) was co-transfected to normalise the data. Each luciferase assay included non-transfected cells and empty-vector-transfected cells as negative controls. After 24 h, the transfected cells were treated according to the Dual-Luciferase Reporter Assay System protocol (Promega), and luciferase activity was measured using a Synergy 2 (BioTek Instruments, Inc., Madison, WI, USA) plate reader. At least three independent transfections were performed (*n* = 3), each in triplicate, to ensure the data reproducibility. The results are reported as the mean of luciferase activity from triplicates, and the standard deviation is shown.

### 2.7. Immunofluorescence and Confocal Microscopy

The ovaries were collected from the P7 *Foxl2* WT and *Foxl2^−/−^* mice and fixed in Histochoice (Amresco, Solon, OH, USA) at room temperature for 4 h. After deparaffinisation, sections were treated with 3% H_2_O_2_ for 1 h, then unmasked using Citrate Buffer solution 1× (#AP-9003-500, Lab Vision^TM^, Thermo Fisher Scientific Inc., Waltham, MA, USA) and 0.01 M EDTA, pH 8. The slides were permeabilised with 0.1% Triton X-100 (Merck), blocked with 3% bovine serum albumin (#05488, Sigma-Aldrich Corp., St. Louis, MO, USA) for 30 min at room temperature, and incubated overnight at 4 °C with primary antibodies. The antibodies used were anti-FOXL2 (#ab5096, 1:25, Abcam, Cambridge, UK), anti-TSC1 (#MBS176028, 1:200, MyBioSource, San Diego, CA, USA), anti-KITL (#bs-0545R, 1:200, Bioss, Woburn, MA, USA), and anti-VASA ((#A21206, BD Pharmingen 560189, 1:100). Secondary antibodies included Alexa Fluor 488 goat anti-rabbit, 1:400 Thermo Fisher Scientific Inc., Waltham, MA, USA), Alexa Fluor 594 goat anti-human (Thermo Fisher #A11014, 1:400) and Alexa Fluor 594 donkey anti-goat (#A11058, 1:500, Thermo Fisher Scientific Inc., Waltham, MA, USA). Immunofluorescence images were acquired using a Leica DMIRE2-TCS-SL Confocal Laser Scanning microscope (Leica Microsystems, Wetzlar, Germany) or with a HiRes CCD camera on a Deltavision system, version 5.10 (DeltaVision, Almere, The Netherlands).

### 2.8. Statistics

For quantitave PCR and luciferase experiments, values were reported as the mean ± standard deviations (SDs) of at least three independent assays. Statistical significance was estimated by Student’s *t*-test and was reported accordingly: ns *p* > 0.05; * *p* ≤ 0.05; ** *p* ≤ 0.01; *** *p* ≤ 0.001; and **** *p* ≤ 0.0001.

Regarding enrichment analyses, we used Metascape automatic settings [35]: *p*-values are calculated based on the cumulative hypergeometric distribution, and q-values are calculated using the Benjamini–Hochberg procedure to account for multiple testing ): *p*-values are calculated based on the cumulative hypergeometric distribution, and q-values are calculated using the Benjamini–Hochberg procedure to account for multiple testings. The kappa scores are used as the similarity metric when performing hierarchical clustering on the enriched terms, and sub-trees with a similarity >0.3 are considered clusters. The most statistically significant term within a cluster is selected to represent it.

## 3. Results

### 3.1. ChIP-Seq: Pathway and Process Enrichment Analysis

**Figure 1:**
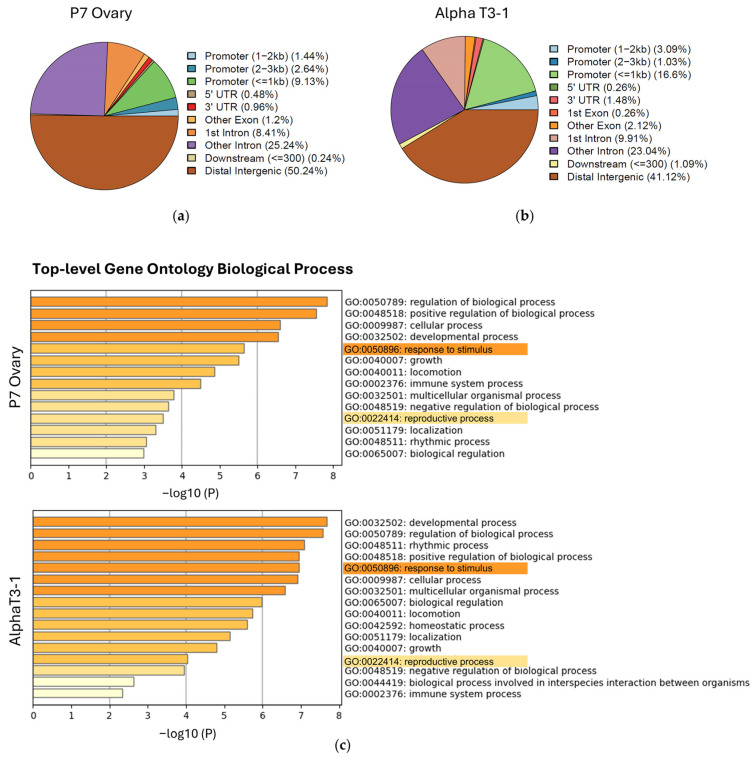
Distribution of ChIP-Seq peaks and enrichment analysis of pathways and processes. (**a**,**b**) Pie charts illustrate the gene feature categories for ChIP-Seq peaks enrichment in P7 ovary and Alpha T3-1 pituitary cells. (**c**) Bar graph of top-level gene ontology biological process enriched terms coloured by *p*-values, across input gene lists from Alpha T3-1 and P7 ovary ChIP-Seq (Supplementary Table S3).

To identify FOXL2 targets, we performed ChIP-Seq experiments using an anti-FOXL2 antibody targeting the C-terminal region in the WT P7 mouse ovaries and in the Alpha T3-1 pituitary cell line, both of which endogenously express FOXL2. ChIP-Seq results were partially reported [28]. Interestingly, based on the sample background represented by the input DNA that has not been immunoprecipitated, we observed (GalSeq analysis) a high relative enrichment of FOXL2 binding at promoter regions (Figure 1a,b). We identified 416 significant bound regions in the ovary and 1554 in the Alpha T3-1 cell line (Supplementary Tables S1 and S2). Fifty-five peaks corresponding to 41 genes overlapped between the two experiments (Supplementary Table S3). For the enrichment analysis we prepared gene lists starting by selecting the narrow peaks from Suplementary Tables S1 and S3 filtered for *p* Value(−log10) > 10 and eliminating duplicated genes (Supplementary Table S4). The most statistically significant enriched terms were “signalling pathways regulating pluripotency of stem cells”, “cellular response to hormone stimulus”, and “canonical Wnt signalling pathway” (Supplementary Figure S2). These pathways are essential in the ovary for embryonic development, follicle development (folliculogenesis), steroid hormone production, and overall female fertility. The enrichment analysis in both the Alpha T3-1 and the ovary revealed that the top-level GO biological processes were “developmental process” and “reproductive process” (Figure 1c), confirming that the ChIP-Seq results are consistent with FOXL2’s known functions. The analysis highlighted, among the terms in the ovary, GO:0070372-regulation of the ERK1/2 cascade and GO:0060395-SMAD protein signalling (Supplementary Figure S3). ERK1/2 and SMAD are important pathways for ovarian development and function: the ERK1/2 pathway can be activated by FSH and IGF1, and SMAD proteins are the downstream signalling molecules of the transforming growth factor beta (TGF-beta) superfamily [38]. In Alpha T3-1, terms of interest included GO:0030111, the regulation of the Wnt signalling pathway, and GO:0032870, the cellular response to hormone stimulus (Supplementary Figure S4). The pathway analysis of the 113 common genes with no shared peaks between the two experiments (different bound region or different peak shape near the same gene) (Supplementary Table S3) revealed the enrichment of the R-MMU-9839406 term related to the regulation of activin signalling by TGFBR3 (Supplementary Figure S5), a process previously linked to POI and ovarian reserve [39,40,41].

Considering the top 100 enriched pathways, TOR signalling, GO:0031929, is enriched (3.859) only in ChIP-Seq from the ovary, −log10 (P) of −2.326.

### 3.2. FOXL2 Binds to a Putative Responsive Element in the 5′ UTR of the Tsc1 Gene

**Figure 2:**
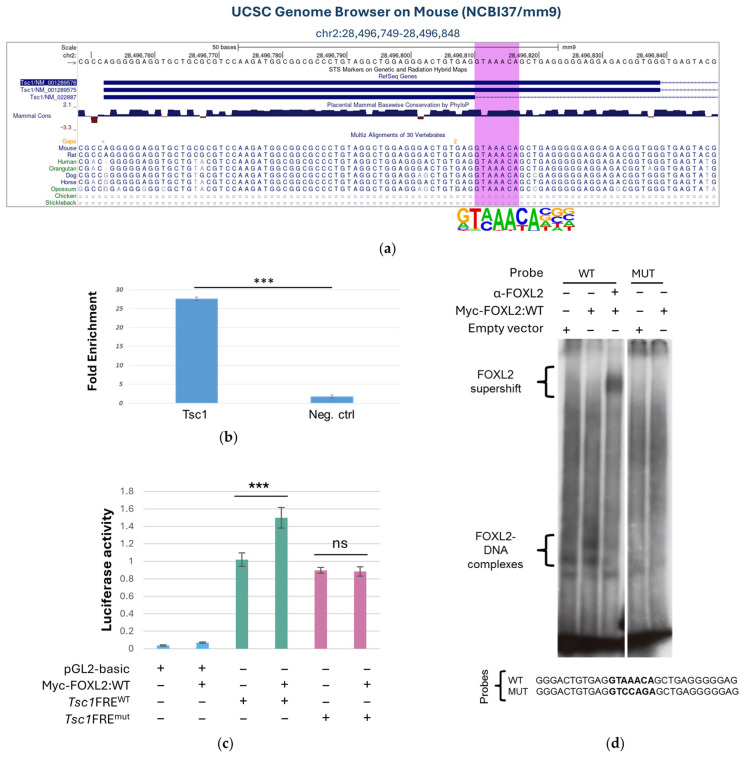
Identification and characterisation of the FOXL2 BS in the 5′ UTR region of the *Tsc1* gene. (**a**) UCSC region showing the FOXL2-bound genomic region (chr2:28496749-28496848, mm9 assembly) identified by ChIP-Seq assays performed in the Alpha T3-1 pituitary cell line and in *Foxl2* WT P7 mouse ovaries. The region is in the 5′ UTR region of the *Tsc1* gene. The bioinformatics analysis identified a putative forkhead transcription factor binding site, GTAAACA, conserved across vertebrates. (**b**) ChIP-qPCR in three independent experiments from P7 ovaries confirms FOXL2 binding to the 5′ UTR region of *Tsc1.* DNA enrichment was consistently observed in the immunoprecipitated samples compared to mock controls. (**c**) Luciferase assay shows that FOXL2 significantly enhances transcription of the luciferase reporter construct, *Tsc1*:FRE^WT^, whereas it is unable to increase transcription of the *Tsc1*:FRE^MUT^ reporter construct. Statistics values are reported as the mean ± standard deviations (SDs) of at least three independent assays. Statistical significance was assessed using Student’s *t*-test and reported accordingly: ns, *p* > 0.05; ***, *p* ≤ 0.001. (**d**) DNA binding in vitro was confirmed by EMSA conducted using protein extracts from Alpha T3-1 cells, either transfected with empty vector or with pCRUZ-Myc-*Foxl2*:WT and 32P-γATP-labelled probes corresponding to the 30 bp WT BS and to the 30 bp MUT probes. FOXL2 binds exclusively to the WT probe, creating a protein–DNA complex. The specific anti-FOXL2 (α-FOXL2) antibody caused a supershift of this complex to a higher molecular weight position, confirming that the shifted band seen in EMSA is due to a specific interaction binding. The original images of EMSA can be found in Supplementary Materials.

Using MEME-ChIP and CisFinder, we previously identified a core consensus FOXL2 binding site (BS), -GTAAACA-, which is mainly located within regions enriched in both ChIP-Seq experiments [28]. To prioritise potential targets, we selected peaks showing binding within 10 Kb upstream of the TSS of known genes and centred on the FOXL2 BS, inferring 15 top genes (Supplementary Table S4). Among them, *Tsc1* was the most notable in both, based on its previously proposed role in ovarian development and follicle maturation [12,14,20]. The second independent analysis by the GalSeq pipeline yielded a *p*-value for the *Tsc1* locus in P7 ovaries that did not meet the formal significance threshold (*p*-value(−log10) > 10). Despite this, the manual inspection of the selected locus by IGV—Integrative Genomics Viewer (https://igv.org/, accessed in 1 July 2025), clearly revealed the presence of an enrichment peak (Supplementary Figure S6). The FOXL2 BS is located within the 5′ UTR of *Tsc1* in a common region (chr2:28496630-28496964, mm9 assembly) identified from two independent ChIP-Seq experiments in *Foxl2* WT P7 mouse ovary and Alpha T3-1 (Figure 2a). FOXL2 binding was confirmed by an independent ChIP-qPCR in P7 ovaries from three mice (*n* = 3), with consistent enrichment observed in the immunoprecipitated samples compared to mock controls (Figure 2b).

Finally, we assessed binding specificity in vitro via Electrophoretic Mobility Shift Assay (EMSA) (Figure 2c). We tested two synthetic 30 bp probes containing either the WT or a mutated consensus motif. In protein extracts from Alpha T3-1 cells transfected with either the empty vector or pCRUZ-Myc-*Foxl2*:WT, FOXL2 specifically bound the 30 bp WT probe but not the MUT probe. Additionally, an anti-FOXL2 antibody caused a shift in the protein–DNA complex to a higher molecular weight, confirming binding (Figure 2c). The binding was abolished upon the addition of a cold WT probe as a competitor but was only partially reduced by a cold MUT probe.).

### 3.3. FOXL2 Binding Is Associated with Tsc1 Promoter Transactivation In Vitro

To investigate FOXL2’s potential involvement in Tsc1 transcriptional regulation, we employed the constructs used by Khatri et al., 2010 [37], which contain 755 bases of the Tsc1 locus, including two FoxO-response elements (FREs), cloned upstream of the firefly cDNA (Tsc1:FRE^WT^ and Tsc1:FRE^MUT^). Our results showed that FOXL2 markedly increased transcription of the Tsc1:FRE^WT^ reporter, but not of the Tsc1:FRE^MUT^ reporter (Figure 2d), further confirming the specificity and functionality of the binding motif in vitro.

### 3.4. Tsc1 Differential Expression in Foxl2 WT Versus Foxl2 Null Ovaries

We examined the microarray data from published studies ([42]; www.ncbi.nlm.nih.gov/geo, accessed via GSE12989 in 1 March 2025) to evaluate *Tsc1* expression in ovaries at prenatal stages (13.5 and 16.5 dpc) and at neonatal stages. *Tsc1* was reduced fivefold in *Foxl2* null ovaries, which appeared histomorphologically normal before birth.

### 3.5. Expression and Localization of TSC1/FOXL2 and KITL/VASA1 in P7 Mouse Ovaries

**Figure 3:**
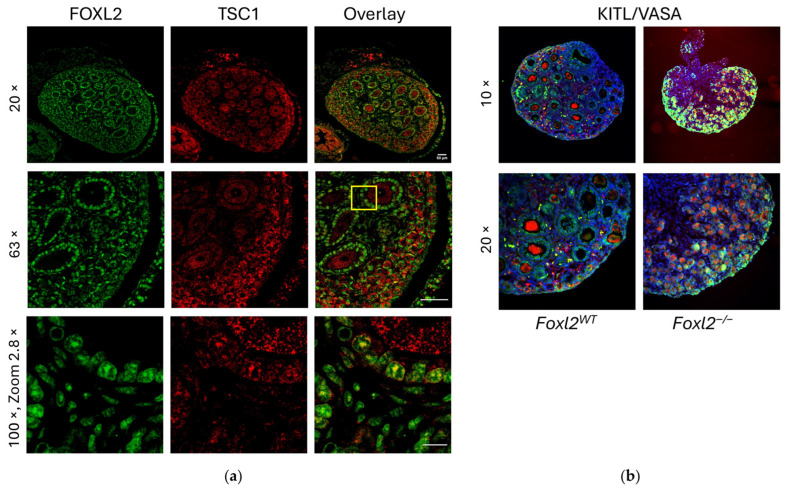
Expression and localization of TSC1/FOXL2 and KITL/VASA1 in P7 mouse ovaries. (**a**) Immunofluorescence was carried out in sections of P7 *Foxl2* WT mouse ovary, using anti-FOXL2 and anti-TSC1 antibodies. FOXL2 is expressed in the GCs of primordial and primary follicles but not in the oocytes. TSC1 is expressed both in the GCs and in the oocytes of maturating follicles. The yellow box highlights the zoomed-in region showing co-localisation at the cell level. (**b**) Immunofluorescence was carried out in sections of P7 *Foxl2* WT and *Foxl2**^−/−^*** mouse ovaries, using anti-KITL and anti-VASA1 antibodies. In blue, nuclear counterstaining performed with DAPI. Immunofluorescence imaging was acquired using a Leica DMIRE2-TCS-SL Confocal Laser Scanning microscope and with a HiRes CCD camera and processed on a Deltavision system version 5.10. Magnification bars indicate 50 μm at 20× and 63×, 10 μm at 100×.

Since ChIP-Seq identified a FOXL2 BS on the *Tsc1* promoter in *Foxl2* WT P7 mouse ovaries, we conducted immunofluorescence studies on the same tissue to analyse TSC1 localisation. We confirmed that TSC1 is expressed in both GCs and oocytes of primordial and primary follicles, particularly in pfGCs in the cortical region, whereas FOXL2 is expressed only in GCs, including pfGCs. The highest magnification shows cellular co-localisation of FOXL2 and TSC1 in a P7 *Foxl2* WT ovary (Figure 3a, 100×).

The mTORC1-KITL cascade in pfGCs awakens dormant oocytes via the KIT-PI3K pathway during follicle activation [12]. Furthermore, the KITL/KIT signalling pathway has a role in somatic cell–germ cell interactions for meiotic entry and progression in the foetal mouse ovary and promotes mTORC/p-S6 signalling by activating the p-Akt pathway [43]). Therefore, we performed an immunofluorescence on the ovaries of *Foxl2* WT and *Foxl2**^−/−^*** P7 mice using anti-KITL and anti-VASA1 antibodies (Figure 3b). VASA is a well-established germ cell marker expressed in oocytes, particularly during early stages of development. In the *Foxl2**^−/−^*** mice at P7 compared with WT, we observe a strong KITL expression in the cortical region, where the primordial follicles reside. During the follicle activation, primordial follicles migrate from the cortex toward the medulla and then complete their development along the perimedullary zone before the ovulation at the ovarian surface. In the *Foxl2**^−/−^*** mice, despite the blockade of follicle maturation and ovarian tissue disorganisation, the activated follicles migrate toward the medulla [5,6].

## 4. Discussion

**Figure 4:**
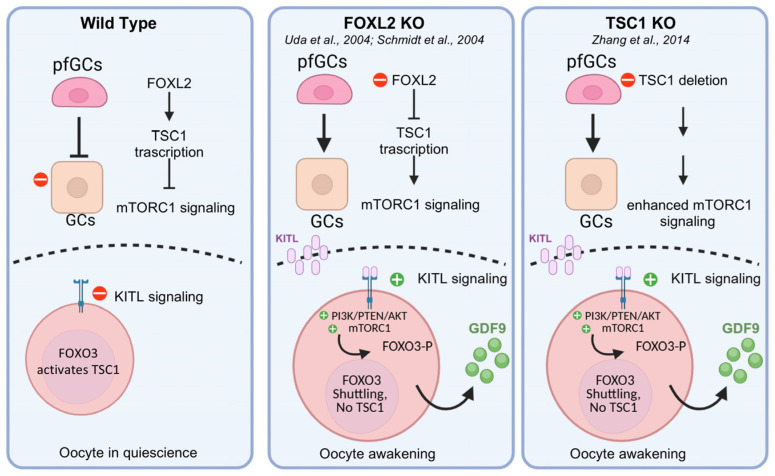
The proposed model for FOXL2 and FOXO3 action in maintaining the ovarian reserve. The main mechanisms of follicle recruitment are described in WT, FOXL2 KO, and TSC1 KO mice models. In WT ovaries, primordial follicles remain quiescent, and pfGCs expressing FOXL2 are flattened. In *Foxl2* KO mutants, the absence of FOXL2 in pfGCs leads to decreased *Tsc1* transcription, which activates mTORC1, promoting primordial follicle activation by increasing KITL. KITL binds cKIT on the oocyte surface, triggering PI3K/Akt signalling, phosphorylating FOXO3, and relocating it to the cytoplasm, thereby abolishing a potential *Tsc1* transcription. The activated PI3K/Akt pathway also inactivates TSC1/2 in oocytes, removing mTORC1 inhibition. GDF9 expression indicates oocyte awakening and follicle activation. Despite high levels of GDF9 and KITL suggesting activation, proliferation remains limited because the lack of FOXL2 prevents pfGCs from transitioning from flattened to cuboidal shape, implying that only mature cuboidal GCs respond to such factors. According to Zhang et al. 2014 [12], *Tsc1* ablation in pfGCs results in mTORC1 hyperactivation and oocyte awakening. When FOXL2 is present, pfGCs differentiate into cuboidal cells, becoming GDF9-responsive, thus promoting follicle growth and maturation [5]. We propose that the master transcription factors FOXL2 and FOXO3 cooperate to maintain follicular quiescence by negatively regulating mTORC1 through TSC1, with FOXL2 acting in pfGCs and FOXO3 in oocytes [6]. The image has been created using Biorender.com.

Our study identifies a conserved FOXL2 binding site at the *Tsc1* locus, suggesting that FOXL2 may participate in its transcriptional regulation. Through the ChIP-Seq and luciferase assays, we found a conserved FOXL2 binding site within the *Tsc1* 5′ UTR/promoter region, along with a putative transactivation role for FOXL2. These findings are consistent with previous reports of FOXL2 occupancy at this locus in foetal ovaries, suggesting a regulatory role that begins in the early development with a time-specific role [44,45]. The biological relevance of such findings in mice, suggesting a potential transcriptional regulation of *Tsc1* by FOXL2, is further underscored by the significant reduction in endogenous *Tsc1* mRNA in *Foxl2* null mice at both the prenatal and neonatal stages [42]. The co-localisation of FOXL2 and TSC1 in GCs supports a model in which FOXL2 maintains primordial follicle dormancy by modulating mTORC1 signalling. Specifically, we hypothesise that a FOXL2-dependent modulation of *Tsc1* expression in pfGCs could serve as a critical brake on follicle recruitment (Figure 4). In the absence of FOXL2, the subsequent decline in *Tsc1* expression likely triggers the following cascade: the activation of mTORC1 signalling within the pfGCs; KITL production and interaction with its receptor on the oocyte surface; the activation of the PI3K/Akt pathway; phosphorylation and cytoplasmic localisation of FOXO3; and the beginning of oocyte growth ([19,46]; Figure 4). Despite this initial activation, the follicles in the *Foxl2* KO models fail to complete development because the somatic cells are dysfunctional and cannot sustain proliferation. This explains the paradox observed in the *Foxl2^−/−^* mice, in which high levels of activation markers such as GDF9 and KITL are present, yet follicle growth remains limited [3,4,6]. It is suggestive that FOXO3 binds the same site and activates the *Tsc1* promoter [37]. Although FOXO3 binding to *Tsc1* was not originally mapped in the ovary, the critical nature of this convergence is evidenced by the phenotypic similarities between various KO models. The specific ablation of *Tsc1* in either pfGCs or oocytes results in the premature activation of the entire primordial follicle pool [12]. This phenotype mirrors that of *Foxo3* ablation, characterised by global follicular awakening and increased mTORC1 activity [12,20]. These matching phenotypes support the idea that the FOXO3→TSC1 regulatory loop also operates in the ovary. Therefore, *Tsc1* appears to be a common target for maintaining quiescence across different ovarian cell lineages.

We propose that FOXL2 and FOXO3 may establish a transcriptional safeguard system through their functional complementarity within the TSC/mTORC1 pathway by regulating *Tsc1*—FOXL2 in pfGCs and FOXO3 in oocytes. In this way, they could govern follicular recruitment and maintain the ovarian reserve (Figure 4). Overall, if our hypothesis is confirmed, it will indicate potential approaches for novel treatments of POI and methods for fertility preservation, including in vitro activation of cryopreserved ovarian tissue and in vitro folliculogenesis. As both FOXL2 and FOXO3 are transcription factors, they are challenging to regulate directly. However, molecules targeting the downstream TSC/mTORC1 pathway —such as rapamycin, an mTOR inhibitor—have shown promise [12,46,47].

Additionally, this finding paves the way for further research into the functions of FOXL2 and TSC1-mediated pathways, including their roles in the pituitary gland.

## 5. Study Limitations

While this study provides compelling evidence of FOXL2 binding at the *Tsc1 locus*, we recognise some limitations that hinder the advancement of this research beyond the proposed mechanistic hypothesis of a regulatory axis involving FOXL2, *Tsc1*, and mTORC1 activity.

Unfortunately, the limited availability of P7 *Foxl2^−/−^* ovaries prevented a direct biochemical measurement of TSC1 protein levels and mTORC1 signalling (e.g., p-S6 or p-4EBP1), which could have helped clarify the functional connection.

In addition, regarding our experimental models, we acknowledge that to better validate physiological relevance, the results from HEK293 cell transactivation assays should have been confirmed in ovarian granulosa cell lines, such as murine KK1, which naturally express FOXL2. However, the experiments we conducted in these cells, although showing a similar trend to those observed in HEK293 cells, did not achieve the same levels of statistical significance. This may be due to regulatory constraints—such as potential promoter saturation and feedback mechanisms—that buffer FOXL2 activity.

## 6. Conclusions

The mTORC1 signalling pathway must be strictly regulated in both oocytes and somatic cells to ensure reproductive longevity. FOXL2, through its observed interaction with the *Tsc1* locus, potentially acts as a somatic gatekeeper of this pathway, ensuring that TSC1 expression is sufficient to prevent premature exhaustion of the ovarian reserve. While further experiments are required to fully clarify the involved mechanisms, such as analysing downstream effectors of the mTORC1 pathway following modulation of FOXL2 expression in mouse models or suitable cell lines, this study provides a foundational framework, although further in vivo studies are required to confirm a causal regulatory relationship.

## Data Availability

The original data presented in the study will be openly available in FigShare DOI:10.6084/m9.figshare.31851823.

## Supplementary Materials

The following supporting information can be downloaded at: https://www.mdpi.com/article/10.3390/biom16040510/s1, The Supplementary figure file contains: Figure S1. ChIP validation by qPCR; Figure S2. Pathway and process enrichment analysis for the 41 genes corresponding to the matched peaks between P7 ovary and pituitary cells; Figure S3. Pathway and process enrichment analysis of the putative FOXL2 target genes in P7 ovary; Figure S4. Pathway and process enrichment analysis for the putative FOXL2 target genes in Alpha T3-1 pituitary cells; Figure S5. Pathway and process enrichment analysis for the putative FOXL2 target genes common to P7 ovary and Alpha T3-1 pituitary cell; Figure S6. Read alignment from ChIP-Seq, in the Tsc1 promoter region. The Supplementary Table file contains: Table S1: P7 Ovary_ All peaks identified by GalSeq analysis. Table S2: Alpha T3-1_ All peaks identified by GalSeq analysis. Table S3: P7 Ovary and Alpha T3-1 peaks selected from ChIP 2016 analysis, with the following criteria: peaks located within 10kb from genes, and centred on the Binding Site (BS). Table S4: Gene lists used for Enrichment analysis, corresponding to peaks from Supplementary Tables S1 and S3, filtered for *p* Value(−log10) > 10, and w/o duplicates. Original images of EMSA can be found in Supplementary Materials.

## Abbreviations

The following abbreviations are used in this manuscript:

POI	primary ovarian insufficiency
pfGCs	primordial follicle granulosa cells
WT	wild-type
KO	knock-out
