# Supplementary material for: Evidence for FOXL2 Association with the Tsc1 Regulatory Region in Mice
Source: Biomolecules. 2026 Mar 29;16(4):510. doi: 10.3390/biom16040510 (PMC13113763; doi:10.3390/biom16040510)
Supplement: Supplementary file 1 [file biomolecules-16-00510-s001.zip › biomolecules-4180611-Supplementary final 0331/biomolecules-4180611-Supplementary/Supplementary Figures_revised.pdf]

Supplementary Figures

In the main file, we focused on the importance of Tsc1 as a putative FOXL2-regulated gene, essential for ovarian function. In the supplementary figures, we report preliminary ChIP validation experiments, the identified peak upstream of Tsc1, and a detailed description of the enrichment analysis performed by Metascape. The figures show the main enrichment results from the gene list identified by ChIP-Seq in the P7 ovary and AlphaT3-1, and from shared peaks between the two experiments. To perform enrichment analysis, we selected only the most significant peaks, using the stringent criteria qValue(−log10) = 6, corresponding to pValue(−log10) of 10.

Figure S1

ChIP validation

(a)

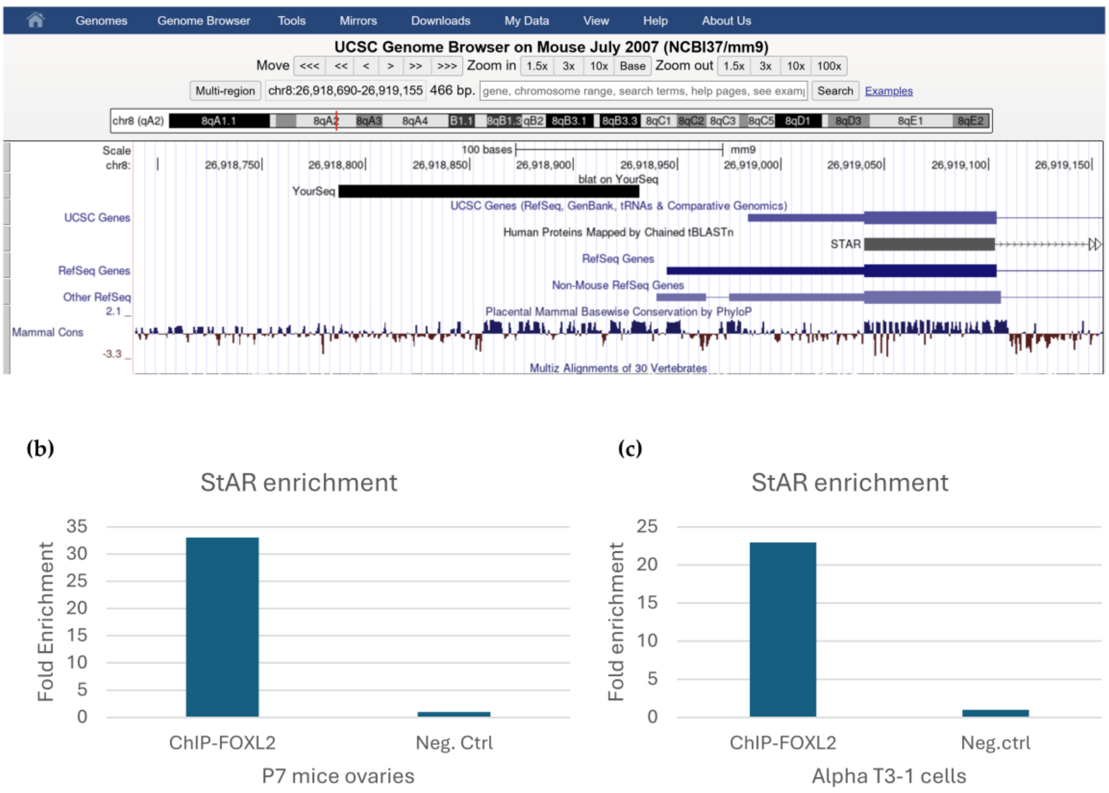

Figure S1. ChIP validation by qPCR.
Before proceeding with sequencing, qPCR was performed to verify enrichment of the known FOXL2-regulated region upstream of the StAR gene. (**a**) The FOXL2-regulated region of StAR, described in Pisarska et al., 2004 [30] and amplified by qPCR as a positive control of ChIP experiments, is shown in the UCSC browser as YourSeq; (**b**,**c**) ChIP-qPCR shows the enrichment of the StAR-bound region in P7 mice ovaries and in AlphaT3-1 cells respectively.

Figure S2

Enrichment analysis for shared peaks

(a)

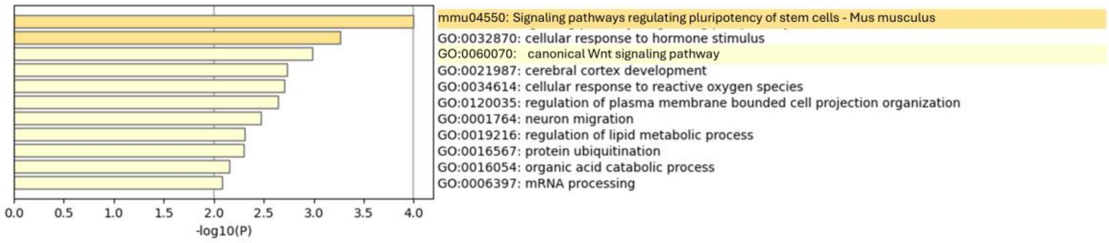

(b)

GO	Category	Description	Count	%	Log10(P)	Log10(q)
mmu04550	KEGG Pathway	Signaling pathways regulating pluripotency of stem cells - Mus musculus (house mouse)	4	10.81	-3.99	0
GO:0032870	GO Biological Processes	cellular response to hormone stimulus	6	16.22	-3.27	0
GO:0060070	GO Biological Processes	canonical Wnt signaling pathway	3	8.11	-2.99	0
GO:0021987	GO Biological Processes	cerebral cortex development	3	8.11	-2.73	0
GO:0034614	GO Biological Processes	cellular response to reactive oxygen species	3	8.11	-2.71	0
GO:0120035	GO Biological Processes	regulation of plasma membrane bounded cell projection organization	6	16.22	-2.64	0
GO:0001764	GO Biological Processes	neuron migration	3	8.11	-2.47	0
GO:0019216	GO Biological Processes	regulation of lipid metabolic process	4	10.81	-2.31	0
GO:0016567	GO Biological Processes	protein ubiquitination	5	13.51	-2.3	0
GO:0016054	GO Biological Processes	organic acid catabolic process	3	8.11	-2.15	0
GO:0006397	GO Biological Processes	mRNA processing	4	10.81	-2.09	0

(c)

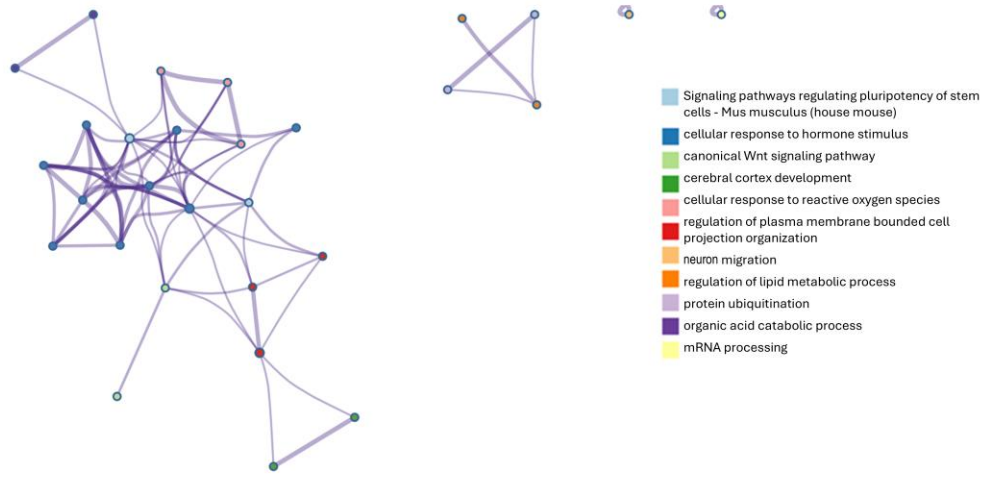

Figure S2. Pathway and process enrichment analysis for the 41 genes corresponding to the matched peaks between P7 ovary and pituitary cells

The figure shows the top 11 clusters with their representative enriched terms. (**a**) Bar graph of the top-level Gene Ontology biological process enriched terms across input gene lists, coloured by p-values. (**b**) Table of the top-level Gene Ontology biological processes. "Count" indicates the number of genes in the user-provided lists that are associated with each ontology term. "%" represents the percentage of all the provided genes that are found in the specific ontology term. "Log10(P)" is the p-value expressed in log base 10. "Log10(q)" is the multi-test adjusted p-value in log base 10. (**c**) Network of the top-level enriched terms coloured by cluster ID, where nodes sharing the same cluster ID are usually close to each other.

Figure S3

Metascape: Enrichment analysis for P7 ovary target genes

(a)

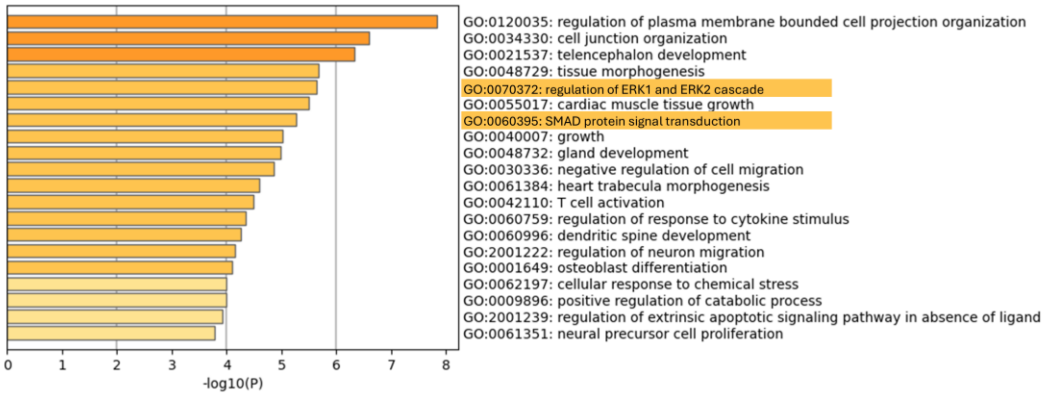

(b)

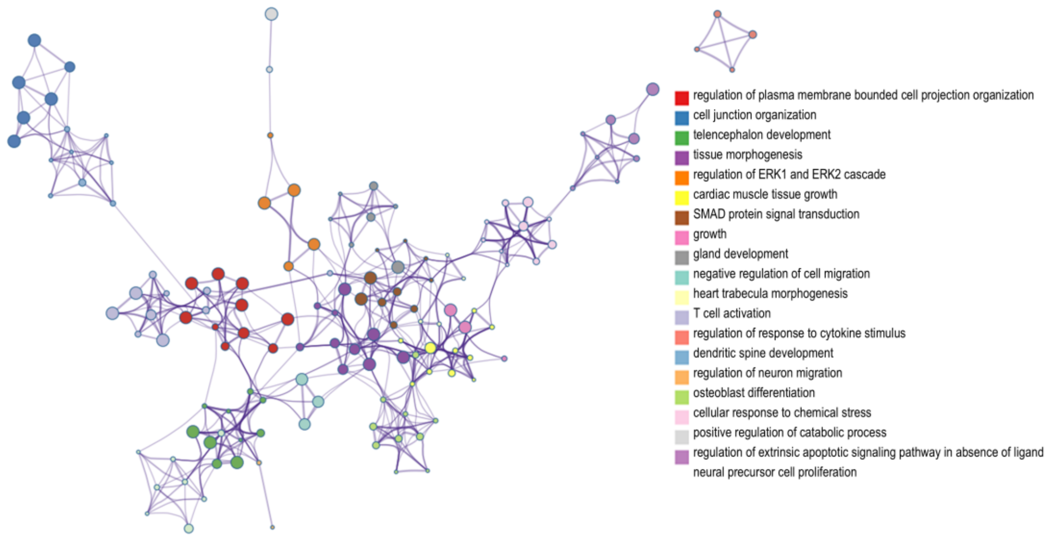

Figure S3. Pathway and process enrichment analysis of the putative FOXL2 target genes in P7 ovary

The figure shows the top 20 clusters, each with a representative enriched term. (**a**) Bar graph of enriched terms, colored by p-values. (**b**) Network of enriched terms colored by cluster ID, where nodes sharing the same cluster ID are usually close to each other.

Figure S4

Metascape: Enrichment analysis for Alpha T3-1 target genes

(a)

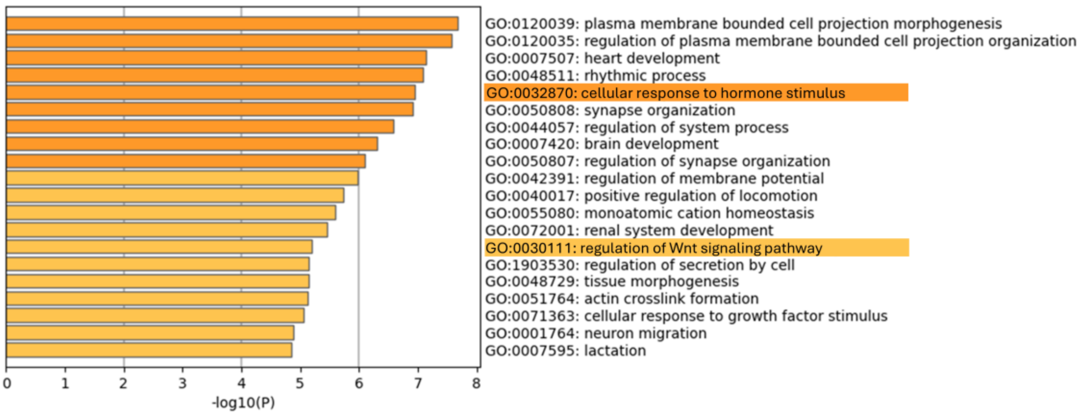

(b)

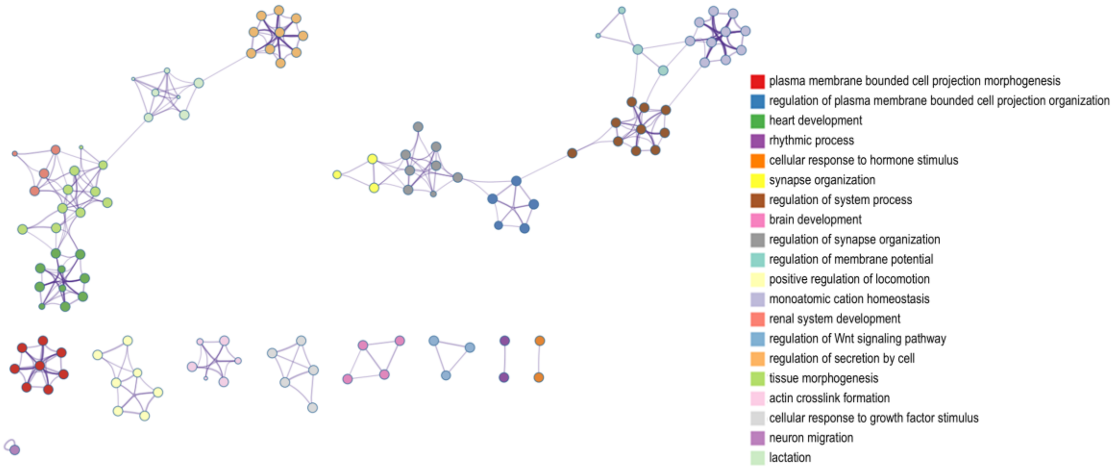

Figure S4. Pathway and process enrichment analysis for the putative FOXL2 target genes in Alpha T3-1 pituitary cells. Top 20 clusters, each with its representative enriched term. (**a**) Bar graph of enriched terms, colored by p-values. (**b**) Network of enriched terms colored by cluster ID, where nodes sharing the same cluster ID are usually close to each other.

Figure S5

Metascape: Enrichment analysis for Common genes (no shared peaks)

(a)

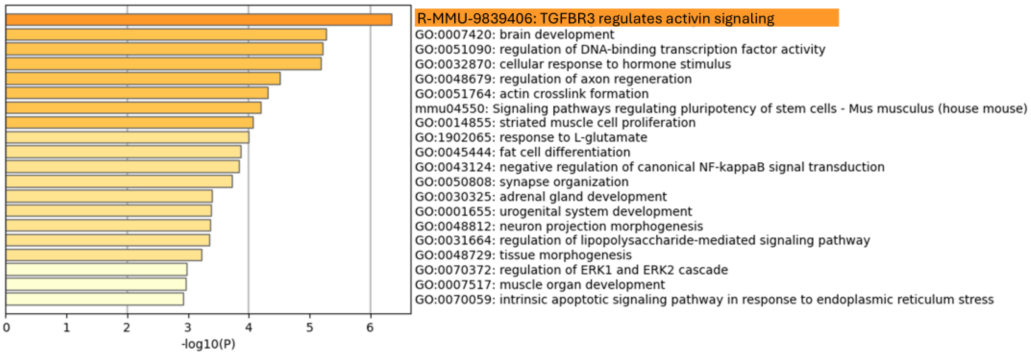

(b)

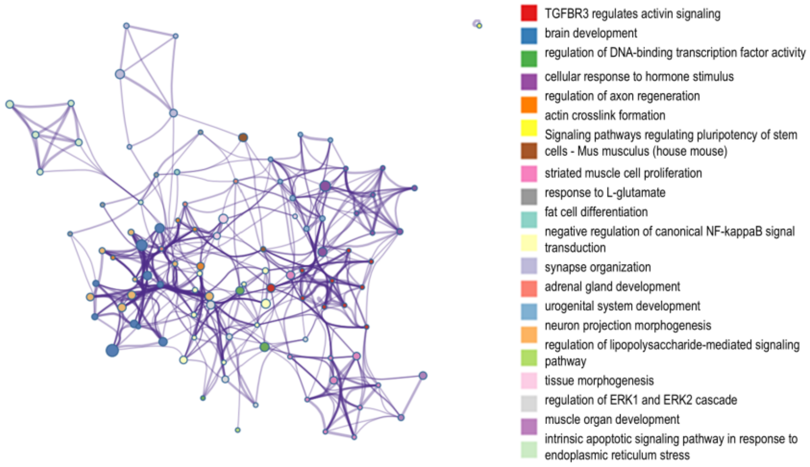

Figure S5. Pathway and process enrichment analysis for the putative FOXL2 target genes common to P7 ovary and Alpha T3-1 pituitary cell

Here, we selected shared regulated genes. Peaks were not always overlapping. The figure shows the top 20 clusters, each with their representative enriched term. (**a**) Bar graph of enriched terms, colored by p-values. (**b**) Network of enriched terms colored by cluster ID, where nodes sharing the same cluster ID are usually close to each other.

Figure S6

(a)

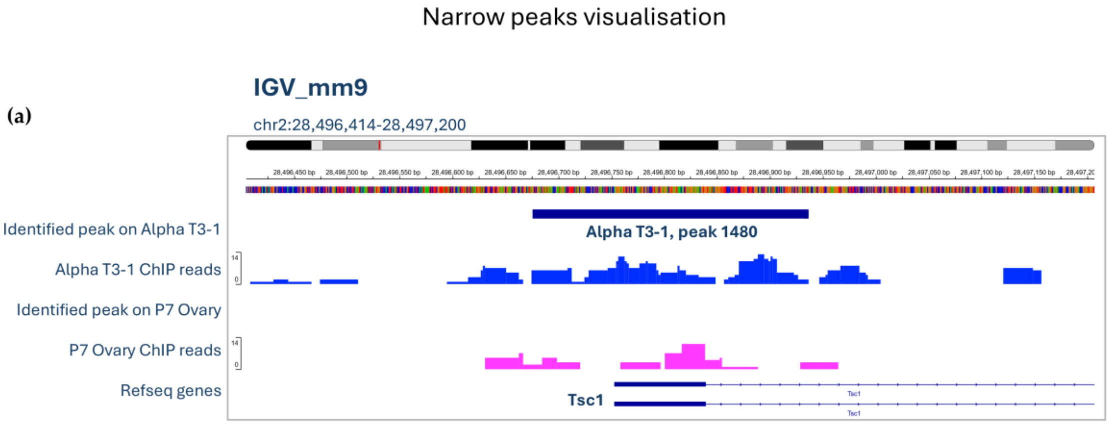

Figure S6. Read alignment from ChIP-Seq, in the Tsc1 promoter region. (**a**) Manual inspection of the selected locus clearly revealed a peak: bigWig files (.bw) loaded into Integrative Genomics Viewer (https://igv.org/app/, accessed in 1 July 2025) show a region bound in both P7 ovary and AlphaT3-1.
